# APTES: a high-throughput deep learning–based Arabidopsis phenotypic trait estimation system for individual leaves and siliques

**DOI:** 10.1007/s42994-025-00239-y

**Published:** 2025-10-31

**Authors:** Ruifang Zhai, Ning Tang, Zhi Liu, Sha Tao, Yupu Huang, Xue Jiang, Aobo Du, Jiashi Wang, Tao Luo, Jinbao Liu, Gina A. Garzon-Martınez, Fiona M. K. Corke, John H. Doonan, Wanneng Yang

**Affiliations:** 1 National Key Laboratory of Crop Genetic Improvement, National Center of Plant Gene Research, Hubei Hongshan Laboratory, Huazhong Agricultural University, Wuhan, 430070 China; 2 Engineering Research Center of Intelligent Technology for Agriculture, Ministry of Education, Huazhong Agricultural University, Wuhan, 430070 China; 3 State Key Laboratory of Crop Stress Adaptation and Improvement, School of Life Sciences, Henan University, Kaifeng, 475004 China; 4 National Plant Phenomics Centre, Institute of Biological, Environmental and Rural Sciences, Aberystwyth University, Aberystwyth, Ceredigion SY233EB UK

**Keywords:** Instance segmentation, High-throughput phenotyping, Arabidopsis, Leaf, Seed pod, Silique

## Abstract

High-throughput phenotyping of growth kinetics and organ size in the model plant *Arabidopsis thaliana* requires rapid and precise methods for trait estimation. To address this need, we developed the Arabidopsis Phenotypic Trait Estimation System, APTES, an open-access, high-throughput program that uses computer vision and deep learning to extract 64 leaf traits and 64 silique traits from photographs. The enhanced segmentation model Cascade Mask Region-based Convolutional Neural Network (Mask R-CNN) achieved precision (measure of positive prediction accuracy), recall (sensitivity in detection), and F1 score values (harmonic mean of precision and recall) of 0.965, 0.958, and 0.961, respectively, for individual leaf segmentation. These metrics demonstrated a consistent improvement of approximately 1 percentage point over the baseline model. For silique segmentation, our enhanced DetectoRS model for silique segmentation attained precision, recall, and F1 scores of 0.954, 0.930, and 0.942, respectively. Notably, precision increased by 1%, while the F1 score improved by 2 percentage points. Trait parameters were automatically calculated with coefficient of determination values for leaf and silique traits ranging from 0.776 to 0.976 and mean absolute percentage error values from 1.89% to 7.90%. We phenotyped 166 Arabidopsis accessions, using APTES, and subjected the resulting values to a genome-wide association study (GWAS), revealing 1,042 single-nucleotide polymorphisms (SNPs) as being significantly associated with 18 leaf and silique traits, and one significant SNP on chromosome 3 linked to silique number. Furthermore, we validated APTES across other public Arabidopsis databases and other plant species, with segmentation results demonstrating its applicability across diverse datasets. In conclusion, APTES is a valuable automated tool for leaf and silique segmentation and trait estimation, which should offer benefits to the broader plant science community.

**Supplementary Information:**

The online version contains supplementary material available at 10.1007/s42994-025-00239-y.

## Introduction

Establishing genotype–phenotype associations in model systems such as Arabidopsis (*Arabidopsis thaliana*) has helped identify desirable agronomic traits and potentially engineer them for transfer into crops, thereby improving environmental adaptation, productivity, and nutritional quality. However, significant challenges remain in gene–trait association studies, particularly owing to limitations in automated phenotyping methodologies. The current limited ability to precisely quantify phenotypic traits and link them to specific genetic loci impedes the establishment of robust genotype–phenotype relationships, leaving gaps in our understanding of the biological mechanisms underlying specific traits. These limitations underscore the critical need for developing more accurate and efficient automated phenotyping methods.

The field of phenomics (Pieruschka and Schurr 2019) has emerged to address the growing challenge of characterizing phenotypic variation across large populations. Central to this effort is the quantification of morphometric traits, such as organ size and shape, which influence plant biology and economically important characteristics. However, the structural complexity and diversity among different species, or across distinct developmental stages within the same species, call for specialized approaches for data acquisition and analysis. Traditional manual measurements of morphological traits (e.g., leaf perimeter, leaf area) are labor-intensive, prone to error, and often destructive in nature (Großkinsky et al. 2015; Pajares 2015), severely limiting their scalability. Recent advancements in automated imaging systems and robotic handling have alleviated bottlenecks in primary data collection in the field and in controlled environments, but the current challenges have shifted to computational analysis, particularly in terms of feature extraction and biological interpretation of complex phenotypic data.

### Computer vision methods in plant phenotyping

Recent advancements in sensor technology and computer science, especially machine learning/artificial intelligence, are rapidly revolutionizing the development of phenomics (Furbank and Tester 2011; Pieruschka and Schurr 2019). Among emerging technologies, computer vision and deep learning methodologies that extract and quantify features from images and videos have shown promise as fast, non-invasive alternatives for measuring plant phenotypic data (Del Valle et al. 2018; Mochida et al. 2019). These breakthroughs overcome many of the limitations of conventional manual measurement methods (Han et al. 2018). However, further acceleration of the development and deployment of these algorithms is required to meet the high accuracy needed for phenotypic data in high-throughput studies.

Image segmentation, which involves dividing an image into several parts, is often a prerequisite for phenotyping at the organ level. Two common tasks, namely semantic segmentation and instance segmentation, can be achieved using a variety of state-of-the-art methods. Semantic segmentation involves the pixel-wise classification of an image into its plant and background pixels (Karthik et al. 2022), whereas instance segmentation identifies and delineates instances of objects such as individual leaves or siliques with precise boundaries. Previous studies have developed and applied various methods to segment plant organs such as Arabidopsis leaves and siliques (Hamidinekoo et al. 2020; Hüther et al. 2020). These methods can be largely divided into three categories:

(1) Thresholding-based segmentation: The greenness of plants can provide an effective approach to extract the plant from its background by performing a color-to-grayscale conversion using different color models (Lu et al. 2022). Color-based algorithms can be implemented using various approaches, such as the excess green (ExG) index, the excess red (ExR) index, excess green minus excess red (EGER), normalized color indices (Awlia et al. 2016), color index of vegetation extraction (CIVE), color indices (Del Valle et al. 2018), modified ExG (MExG), and combined indices, among others (Lu et al. 2022), followed by a thresholding method. The Otsu algorithm has been widely applied to plant image segmentation (Otsu 1979). The proprietary PlantScreen software was developed specifically for Arabidopsis rosettes grown on the Photon Systems Instruments platform, providing binary and Red–Green–Blue (RGB) representations for each plant, followed by morphological and greenness analysis that calculate growth-related parameters on a whole-rosette basis (Awlia et al. 2016). Manual adjustments are often required, and parameters, such as contrast, can be adjusted to enhance the difference between the plant and its background, with segmentation then performed on such contrast-enhanced images (Lu et al. 2022). The PlantScreen image analysis pipeline was used to quantify shape and size variation in a large Multiparent Advanced Generation Inter-Cross (MAGIC) mapping population in Arabidopsis and dissect the genetic architecture underlying rosette-related traits (Morón-García et al. 2022). For siliques, the Otsu algorithm was implemented for segmentation, and a cutting method was proposed based on concave point extraction and matching to deal with overlapping siliques, resulting in processed images acceptable for phenotype estimation (Liu et al. 2020). In summary, thresholding-based image segmentation can be easily implemented, with the segmentation result illustrated mostly as a binary image, where pixels with high green-scale values are deemed as plants, whereas the other pixels are considered as background, representing a typical example of semantic segmentation.

(2) Machine learning–based image segmentation: This method offers significant advantages over traditional thresholding-based segmentation, which can be affected by factors, such as background complexity, reflective surfaces, and varying light conditions (Hamuda et al. 2016). Thresholding methods face particular difficulties when plants exhibit color variations, such as across developmental stages or under stress conditions, or when leaves overlap within an individual plant or when plant populations form overlapping canopies. Machine learning can address these issues to a certain extent using training datasets to learn how to label features (Tian et al. 2019). Feature extraction from the training dataset allows a classification model to be created. This trained classifier can then predict the probability that an image segment is or is not part of the feature (e.g., a leaf or a silique), which can be followed by a segmentation procedure. This approach can be implemented using algorithms such as *K*-means clustering or support vector machine (SVM) (Singh et al. 2016), among others. Phenotiki is an example of an open software and hardware system developed to segment and count Arabidopsis leaves using machine learning algorithms (Minervini et al. 2017).

(3) Deep learning–based image segmentation: This method has emerged as a robust approach for accurately and efficiently segmenting plant organs with high throughput. Traditional thresholding or machine learning–based segmentation methods may struggle to accurately handle the variability present across plant materials. Deep learning models, representing a subset of machine learning, offer superior flexibility and performance but may require a larger number of labeled images as a training dataset. Convolutional neural networks (CNNs) employ sliding windows of varying sizes to scan across the image to automatically capture local morphological features in images (Huang et al. 2024). The hierarchical architecture of CNNs, comprising convolutional layers, pooling layers, and fully connected layers, progressively integrates multiscale representations from pixel level to organ level, enabling feature extraction from microscopic to macroscopic levels. Various CNN-based networks, such as Fully Convolutional Network (FCN) (Long et al. 2015), U-Net (Ronneberger et al. 2015), SegNet (Badrinarayanan et al. 2017), and DeepLabV3 (Chen et al. 2018), have been successfully applied to image semantic segmentation tasks. CNNs such as Mask region-based CNN (Mask R-CNN) (He et al. 2017), Cascade Mask R-CNN (Cai and Vasconcelos 2018), Hybrid Task Cascade (HTC) (Chen et al. 2019), and Segmenting Objects by Location (SOLO) (Wang et al. 2020), excel at image instance segmentation tasks.

Usually, computer vision networks produce reliable and accurate segmentation results across a wide range of applications, relying heavily on a large number of labeled training images (Pound et al. 2017). Therefore, the construction of datasets is of the utmost importance. Benchmark datasets for Arabidopsis include the Plant Phenotyping Datasets provided by the International Plant Phenotyping Network (IPPN) (Minervini et al. 2016; Schaar et al. 2014), Arabidopsis Dataset (Hüther et al. 2020), Multi-Modality Plant Imagery Dataset (MSU-PID) (Cruz et al. 2016), and the Arabidopsis full-growth dataset (Morón-García et al. 2022). A series of deep learning–based studies have been conducted on these datasets and proprietary datasets. A deep plant phenomics platform using CNN architecture was developed for counting leaves, classifying mutants, and plant age estimation using the Plant Phenotyping Datasets (Ubbens and Stavness 2017; Ubbens et al. 2018). The Eff-U-Net +  + framework (Bhagat et al. 2022), featuring enhanced encoder-decoder pathways and hierarchical feature integration, achieved state-of-the-art performance in leaf segmentation and counting tasks on multiple phenotyping benchmarks. The ARADEEPOPSIS software used DeepLabV3 + for semantic segmentation of top-view images to classify rosettes and measure traits, such as total area and color (Hüther et al. 2020). A patch-based classification and localization deep learning framework, DeepPod, was developed for silique detection and counting (Hamidinekoo et al. 2020). YOLO v8 and Mask R-CNN were employed for pod segmentation and calculations of phenotypes in rapeseed (*Brassica napus*) (Wang et al. 2023).

### Challenges in Arabidopsis phenotyping

Current whole-plant semantic segmentation techniques provide fundamental phenotypic data, but exhibit significant limitations in achieving precise organ-level quantification for the leaves and siliques of Arabidopsis plants. Existing methodologies are constrained by three critical limitations: First, specialized tools capable of concurrently achieving high-precision segmentation for both Arabidopsis leaves and siliques are lacking, particularly under conditions of organ occlusion or complex backgrounds where conventional image processing approaches (e.g., threshold-based segmentation and edge detection algorithms) perform poorly. Second, current solutions fail to fully exploit recent advancements in deep learning, predominantly relying on traditional computer vision algorithms that cannot localize precise organ boundaries. Third and most critically, prevailing tools suffer from substantial deficiencies in practical applicability and deployment efficiency: these deficiencies manifest as cumbersome operational procedures requiring specialized programming expertise, absence of user-friendly graphical interfaces, lack of high-throughput capability, and poor system scalability. To address these issues, we developed the Arabidopsis Phenotypic Trait Estimation System (APTES), an accurate, robust, automatic, and high-throughput analytical tool for extracting organs, including leaves and siliques, from diverse input images and estimating corresponding traits from the segmented organs. APTES is an easily accessible, executable tool with a graphical user interface (GUI) called aptes.exe, which can be at (https://drive.google.com/drive/folders/1i9IariiIrxuFtVIaRiaIzqvb8Gfg3xTc) or http://plantphenomics.hzau.edu.cn/usercrop/Rice/download. Although it was initially built around Arabidopsis and data derived from mechanized phenotyping platforms, APTES can accommodate different species with a rosette growth habit, grown under various conditions.

## Results

### The APTES pipeline for high-throughput Arabidopsis phenotyping

**Fig. 1:**
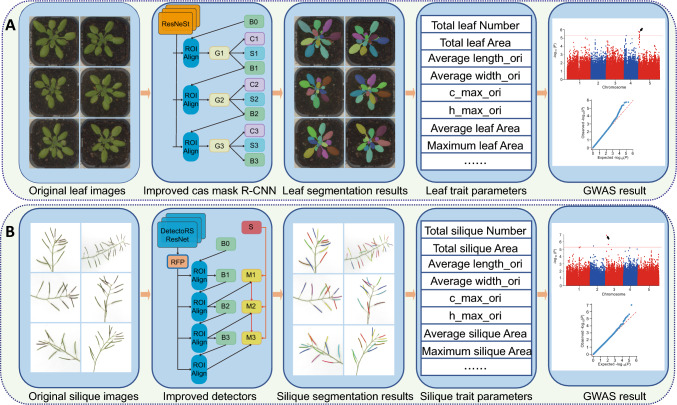
Pipeline behind the Arabidopsis Phenotypic Trait Estimation System (APTES). **A** For leaf analysis, the pipeline starts with leaf segmentation using an Enhanced Cascade Mask R-CNN. Single or multiple leaf images are uploaded to APTES, which produces segmentation results and measures trait parameters for each leaf. These results can be used for GWAS. **B** For silique analysis, the pipeline segments images of siliques using an improved DetectoRS. Individual or multiple images of siliques on branches are uploaded to APTES, which generates segmentation results and measures trait parameters for each silique. These results can be used for GWAS

In this study, we integrated a deep learning approach with computer vision for the segmentation of Arabidopsis organs, leading to the development of the APTES pipeline. The pipeline combines image segmentation with measurements of phenotypic parameters to support high-throughput phenotyping. The segmentation of individual leaves was accomplished using an improved Cascade Mask R-CNN model, and the segmentation of individual siliques from stems and branches was performed with an enhanced DetectoRS model. Users can upload images of leaves or siliques to the pipeline, which automatically generates the segmentation results for individual leaves or siliques. The phenotypic parameters for each leaf or silique are also computed automatically. The segmented results and phenotypic traits can be exported to folders on the user’s local computer. Figure 1 illustrates the entire pipeline. The list of 64 leaf traits and 64 silique traits extracted from the images is provided in Table 1, with detailed explanations provided in Table S1.

**Table 1:** Trait calculation methods

Trait classification	Trait definition	Trait name
Morphological traits	Average leaf perimeter from a single Arabidopsis plant	Average Perimeter
	Maximum leaf perimeter from a single Arabidopsis plant	Maximum Perimeter
	Standard deviation (SD) of leaf perimeter from a single Arabidopsis plant	STD_Perimeter
	Coefficient of variation (CV) of the perimeters for all leaves from a single Arabidopsis plant	CV_Perimeter
	Average leaf roundness from a single Arabidopsis plant	Average Roundness
	Maximum leaf roundness	Maximum Roundness
	SD of leaf roundness	STD_Roundness
	CV of the roundness for all leaves	CV_Roundness
	Leaf number from a single Arabidopsis plant	Total Leaf Number
	Total area for all leaves from a single Arabidopsis plant	Total Leaf Area
	Average leaf area from a single Arabidopsis plant	Average Leaf Area
	Maximum leaf area from a single Arabidopsis plant	Maximum Leaf Area
	SD for leaf area from a single Arabidopsis plant	STD_Area
	CV for leaf area	CV_Area
	Average leaf length from a single Arabidopsis plant	Average length_ori
	Average leaf width from a single Arabidopsis plant	Average width_ori
	Maximum length of the orthogonal bounding rectangle	c_max_ori
	Maximum width of the orthogonal bounding rectangle	h_max_ori
	Average leaf length from a single Arabidopsis plant	Average Length
	Maximum length of the minimum bounding rectangles for all leaves from a single Arabidopsis plant	Maximum Length
	SD of the length of the minimum bounding rectangles for all leaves from a single Arabidopsis plant	STD_Length
	CV of the length of the minimum bounding rectangles for all leaves from a single Arabidopsis plant	CV_Length
	Average leaf width from a single Arabidopsis plant	Average Width
	Maximum width of the minimum bounding rectangle for all leaves from a single Arabidopsis plant	Maximum Width
	SD of the width of the minimum bounding rectangles for all leaves from a single Arabidopsis plant	STD_Width
	CV of the width of the minimum bounding rectangles for all leaves from a single Arabidopsis plant	CV_Width
	Average area of the minimum bounding box for all leaves from a single Arabidopsis plant	Average MBB_Area
	Maximum area of the minimum bounding rectangle for all leaves from a single Arabidopsis plant	MBB_Area
	SD of the area of the minimum bounding rectangles for all leaves from a single Arabidopsis plant	STD_MBB_Area
	CV of the area of the minimum bounding rectangles for all leaves from a single Arabidopsis plant	CV_MBB_Area
	Ratio of the average leaf area to the area of the minimum bounding Rectangles for all leaves from a single Arabidopsis plant	Average Area/MBB_Area
	SD of average leaf area to the area of the minimum bounding rectangles for all leaves from a single Arabidopsis plant	STD_Area/MBB_Area
	Ratio of average perimeter to the average leaf area from a single Arabidopsis plant	Average Perimeter/Area
	Ratio of STD_Perimeter to mean leaf area from a single Arabidopsis plant	STD_Perimeter/Area
	Area of the convex hull = area of the smallest convex polygon for the leaves from a single Arabidopsis plant	Convex Hull Leaf Area
Color traits	Mean value of the red channel for all leaves from a single Arabidopsis plant	R_Ratio_mean
	SD of the red channel for all leaves from a single Arabidopsis plant	STD_R_mean
	Mean value of the green channel for all leaves from a single Arabidopsis plant	G_Ratio_mean
	SD of the green channel for all leaves from a single Arabidopsis plant	STD_G_mean
	Mean value of the blue channel for all leaves from a single Arabidopsis plant	B_Ratio_mean
	SD of the blue channel for all leaves from a single Arabidopsis plant	STD_B_mean
	Proportion of the green component from a single Arabidopsis plant	G_All_mean
Texture traits	Seven numerical values derived from the geometric moments of an image	Hu_moment1–Hu_moment7
	Fifteen gray-level co-occurrence matrix texture-related traits of an image	Gray-Gradient Co-occurrence Matrix

### Advanced models for leaf segmentation and phenotypic trait estimation

**Fig. 2:**
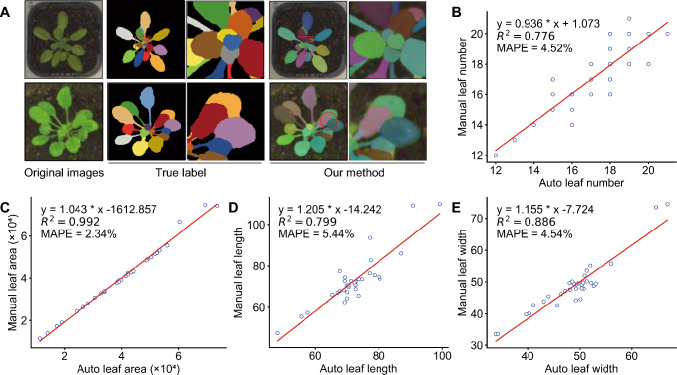
Results of leaf instance segmentation and performance evaluation of trait extraction. **A** Typical examples of results from leaf instance segmentation. From left to right: original leaf images, true labels for individual leaves, and the segmentation results of leaf instances after the improved Cascade Mask R-CNN algorithm. **B-E** Performance evaluation of trait extraction with respect to leaf number (B), leaf area, with values in square pixels (C), leaf length, with values in pixels (D), and leaf width, with values in pixels (E). Each scatterplot shows the relationship between traits measured by the automated pipeline (*x*-axes) and manually (*y*-axes). The linear regressions, their coefficients of determination (*R*^2^), and the mean absolute percentage error (MAPE) values are also shown

How accurate the automatic estimation of phenotypic parameters is for individual leaves heavily depends on the performance of instance segmentation models. Because of its advantages of high accuracy and efficient pixel-level instance segmentation, we chose Mask R-CNN as the foundational model for leaf segmentation. Thus, we enhanced the Cascade Mask R-CNN model by replacing the backbone network from a residual neural network (ResNet) (He et al. 2016) with a residual split-attention network (ResNeSt) (Zhang et al. 2022) and refined the region proposal network (RPN) layer using generic region of interest extraction (GRoIE). ResNeSt employs split-attention mechanisms to enhance multi-scale feature representation, while GRoIE dynamically refines region proposals through deformable convolutions for occluded leaf segmentation. These changes resulted in precise leaf instance segmentation. For model training, we employed two data augmentation techniques on an initial set of 250 images, resulting in 4,250 augmented images. 35 original images were reserved for model testing. Examples of leaf instance segmentation results are presented in Fig. 2A, with each segmented leaf shown in a different color. The segmentation algorithm successfully identified individual leaves, including newly emerged leaves, as delineated by the rectangular bounding box. As marked by the elliptical region in the figure, our method successfully segmented partially occluded leaves and effectively separated two adherent leaves in the image.

We assessed the segmentation accuracy of our pipeline using three metrics: precision, recall, and F1 score (harmonic mean of precision and recall), which achieved values of 0.965, 0.958, and 0.961, respectively. Additionally, in terms of leaf counting, the average difference in count (DiC) between the predicted and ground truth values for all test images was 0.758. The manually quantified ground-truth leaf count was 607, while the predicted count reached 608. Thus, the error for any single image was less than one leaf on average. As another method to compare the automatically segmented results to manual measurements from the same images, we calculated the coefficient of determination (*R*^2^) and mean absolute percentage error (MAPE). Specifically, the *R*^2^ values for leaf number, leaf area, leaf length, and leaf width were 0.776, 0.992, 0.799, and 0.886, respectively. The MAPE values for the same parameters were 4.52, 2.34, 5.44, and 4.54%, respectively (see Fig. 2B for details).

### Advanced models for silique segmentation and phenotype estimation

**Fig. 3:**
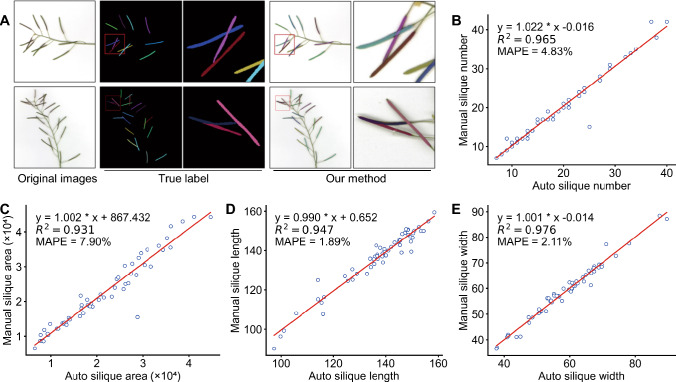
Results of silique instance segmentation and performance evaluation of trait extraction. **A** Representative results of silique instance segmentation. From left to right: original image of siliques on branches at maturity, annotation for individual siliques, and segmentation results using the improved DetectoRS algorithm. **B-E** Performance evaluation of trait extraction with respect to silique number (**B**), silique area, with values in square pixels (**C**), silique length, with values in pixels (**D**), and silique width, with values in pixels (**E**). Each scatterplot shows the relationship between traits measured by the automated pipeline (*x*-axes) and manually (*y*-axes). The linear regressions, their coefficients of determination (*R*^2^), and the mean absolute percentage error (MAPE) values are also shown

Leaf size and number reflect vegetative biomass, and reproductive fitness can be estimated by seed production. The number and size of siliques serve as indicators of seed number and total seed weight (Alonso-Blanco et al. 1999). Although real-time recording of the growth from flowering shoots and silique production is technically possible, extracting biologically meaningful metrics from such an intricate three-dimensional morphology is challenging and hard to scale up for large datasets. Consequently, we used post-harvest 2D scanned images that preserve most of the pertinent biological information from the stems harboring siliques (Tanabata et al. 2012). Instance segmentation of siliques then allows the measurement of morphometric parameters, such as silique length and width. To this end, we developed a silique instance segmentation model using the enhanced DetectoRS network incorporating group normalization (GN) and weight standardization (WS). The combination of GN and WS was specifically designed to mitigate the adverse effects of small batch sizes in experiments and to enhance instance segmentation performance. Figure 3B shows two examples of silique images that were precisely segmented by the model. For training of the instance segmentation model, we used a dataset comprising 1,840 images, which was augmented to a total of 2,357 images. For evaluation of model performance, we used 57 original non-augmented images. The model achieved precise segmentation of siliques, even in complex scenarios where siliques overlapped to different levels (Fig. 3A).

The objective of this study was to develop a pipeline that would be both highly accurate and user-friendly for segmenting individual organs and measuring phenotypic traits. We, therefore, evaluated the accuracy of deep learning segmentation models and the extracted phenotypic parameters. The precision, recall, and F1 score were 0.954, 0.930, and 0.942, respectively. Furthermore, the average DiC for silique number across all test images was 0.857, indicating a close match between the segmented and actual silique counts, with an average error below one silique per image. Additionally, the *R*^2^ values for silique number, area, length, and width were 0.965, 0.931, 0.947, and 0.976, respectively, whereas the corresponding MAPE values were 4.83, 7.90, 1.89, and 2.11% (Table 2). These results demonstrate the high accuracy of the proposed pipeline.

**Table 2:** Performance of the original and improved segmentation models (Bold denotes the results from our method)

Datasets	Method	Precision	Recall	F-score
Leaf	Cascade Mask R-CNN	0.956	0.947	0.951
	Cascade Mask R-CNN + GRoIE	**0.965**	**0.958**	**0.961**
Silique	DetectoRS	0.944	0.930	0.921
	DetectoRS + (GN + WS)	**0.954**	**0.930**	**0.942**

### GWAS for leaf and silique traits using the phenotypes measured by the APTES pipeline

**Fig. 4:**
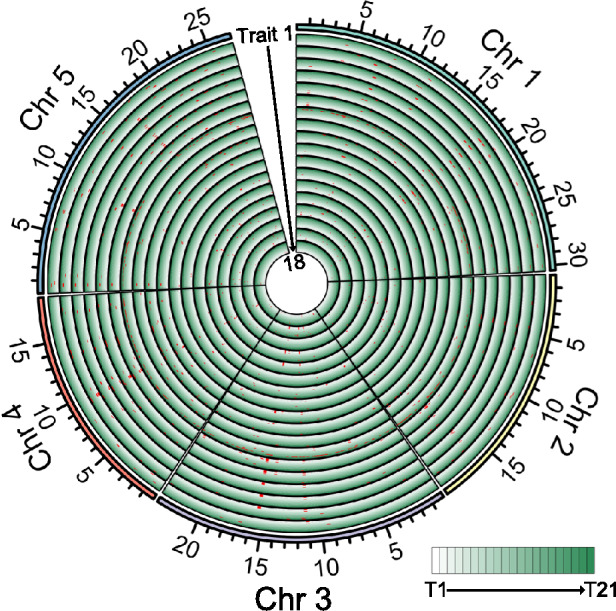
Integrated map of genomic regions associated with 18 leaf-related traits extracted by the APTES pipeline for an *Arabidopsis* population of 162 accessions. The numbers on the circular chromosome tracks indicate genomic positions in megabases (Mb). Traits 1 to 18 represent Average_Area_MBB_Area (1), Average_Leaf_Area (2), Average_length_ori (3), Average_Length (4), Average_MBB_Area (5), Average_Perimeter_Area (6), Average_Perimeter (7), Average_Roundness (8), Average_width_ori (9), Average_Width (10), B_Ratio_mean (11), G_All_mean (12), G_Ratio_mean (13), Maximum_Leaf_Area (14), MBB_Area (15), R_Ratio_mean (16), Total_Leaf_Area (17), and Total_Leaf_Number (18). Trait definitions are provided in Table 1. Each circle covers all 21 time points for each trait, as shown by the color gradient from light to dark

Typically, the integration of phenotypic data with genetic information helps elucidate potential genetic determinants. To evaluate the effectiveness of the APTES pipeline, we grew plants from a set of 166 Arabidopsis accessions from the RegMap panel and phenotyped them over time using the APTES pipeline. We then used all collected phenotypic data as input to identify genetic associations through GWAS on the GWAS-Portal platform, using 250,000 single-nucleotide polymorphisms (SNPs) (Horton et al. 2012). This analysis revealed a total of 1,042 SNPs significantly associated with 18 leaf-related traits across the 21 time points in the Arabidopsis population (Fig. 4 and Table S2). We observed a substantial overlap across genomic regions related to the same and to different traits over time, which may be partly attributed to the high correlations among many of these traits.

**Fig. 5:**
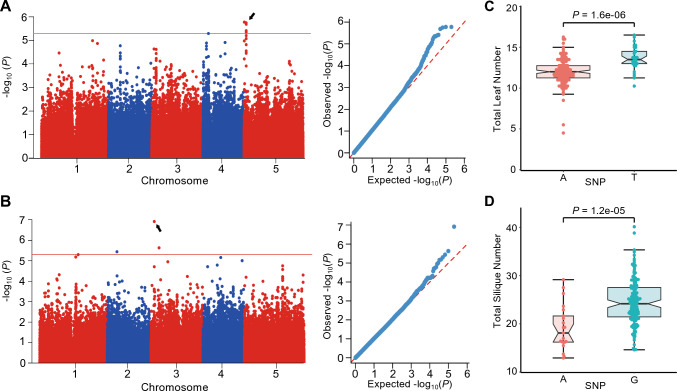
Genome-wide association study (GWAS) of silique and leaf traits collected by APTES. **A**, **B** Manhattan plots (left) and quantile–quantile plots (right) of the association results for leaf number (**A**) or silique number (**B**). **C** **D** The phenotypic data for leaf number (**C**) split by accessions carrying the SNP-A and SNP-T, and distribution of silique number (**D**) split by accessions carrying SNP-A or SNP-G in *OFT1* (*At3g05320*)

We identified a genomic region highly associated with leaf number on chromosome 5 (Fig. 5A, C). Additionally, the improved DetectoRS in APTES enabled accurate phenotyping of silique-related traits in the accessions. An analysis of silique number per plant, conducted using an accelerated mixed-model algorithm, revealed a significantly associated SNP, with A or G as the possible genotypes, on chromosome 3 (Fig. 5B). Accessions carrying SNP-A had fewer siliques than those with SNP-G (Fig. 5D). The significant SNP for silique number was located near the gene *O-FUCOSYL TRANSFERASE 1* (*OFT1*; At3g05320). Notably, *OFT1* was previously reported to encode a Golgi-localized protein, and the *oft1* mutant showed defects in silique development, with lower seed set and lower fertility (Smith et al. 2018). These findings suggest that *OFT1* might be a potential candidate gene contributing to the natural variation of silique number observed in Arabidopsis. The substrate specificity and biochemical mechanisms of OFT1 in the regulation of seed pod development remain to be explored. Nevertheless, our findings substantiate the feasibility of utilizing APTES for elucidating the genetic basis of silique-related traits.

### Time efficiency of the APTES pipeline

The deep learning models in this study were trained and tested on a server running the Ubuntu 20.04 operating system. The server was equipped with an AMD 5800X processor, 64 GB of random-access memory (RAM), and an NVIDIA GeForce RTX 3090 GPU with 24 GB of video RAM (VRAM). The relevant algorithms were implemented using the OpenMMLab framework and the Python 3.7 programming language within a Conda development environment. Detailed hardware and software configuration parameters are provided in Tables S3 and S4. To assess the computational efficiency of the improved models, we processed test images of leaves and siliques using the configurations outlined in the implementation details (see Methods section for further details). For the instance segmentation based on the improved Cascade Mask R-CNN framework, we tested 35 images of rosettes from Arabidopsis plants, with the entire pipeline completing all tasks in 5.2 s, averaging 0.15 s per image (ranging from 80 to 280 KB in size). For siliques, we tested 57 images featuring multiple siliques, which took a total of 16.3 s for the entire processing sequence, with an average processing time of 0.29 s per image. The primary factor contributing to the time discrepancy between leaf and silique image processing is likely the difference in image size and model complexity. In this study, the average size of an input image for leaves was about 200 KB, and around 2 MB for a typical silique image. We have implemented and verified the compatibility of smartphone-acquired images with our analytical pipeline, confirming result validity. Additionally, we evaluated the time efficiency for processing images of varying sizes, with detailed metrics presented in Table S5. This adaptability underscores the robustness of our APTES pipeline in handling images of varying qualities and resolutions, offering accessibility for broader applications beyond controlled experimental settings.

## Discussion

### Comparison to other methods

**Fig. 6:**
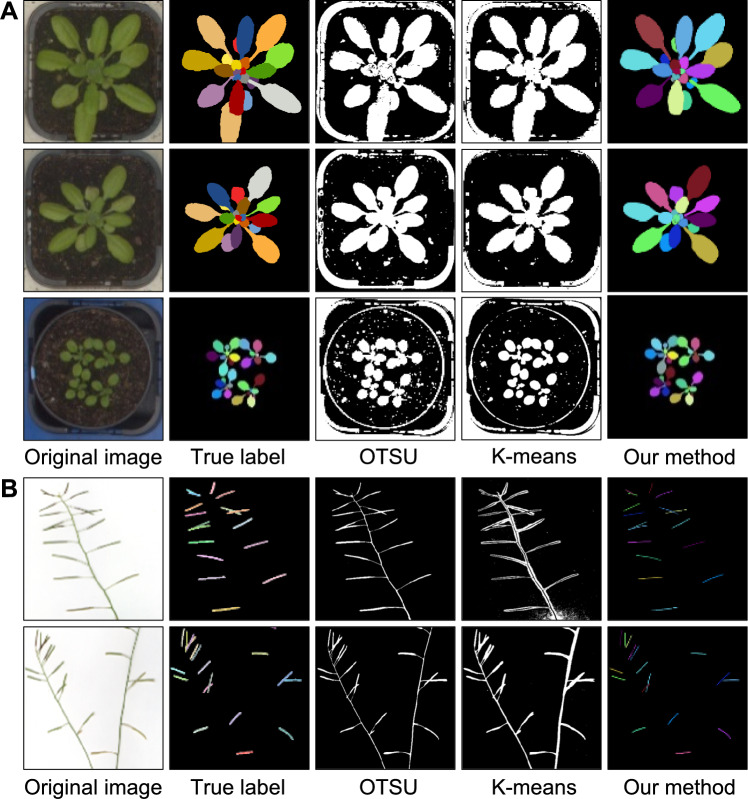
Segmentation results for leaf and silique images using conventional algorithms and our proposed method. **A** Results of leaf segmentation. From left to right: original leaf image, true label for individual leaves, segmentation result with the Otsu algorithm, segmentation result with the *K*-Means clustering algorithm, and our method. **B** Results of silique segmentation. From left to right: original silique images, true label for individual siliques, segmentation result with the Otsu algorithm, segmentation result with the *K*-Means algorithm, and our method

We compared our model to conventional approaches, specifically the Otsu (Otsu 1979) and *K*-Means algorithms, using three randomly chosen images of leaves and two randomly chosen images of siliques (Fig. 6). For leaf images, the Otsu algorithm incorrectly segmented background pixels as parts of plants. Although the *K*-Means algorithm performed better than the Otsu algorithm, it still misclassified some background pixels as plant, requiring additional processing to accurately identify individual plant organs. For silique images, the two conventional methods tested failed to differentiate between stems and siliques, grouping them together as a single object. This inability to distinguish stems from siliques made the segmented results unsuitable for accurate silique counting and trait analysis. By contrast, our APTES pipeline successfully achieved instance segmentation of individual leaves and siliques as distinct objects, each uniquely colored to facilitate identification. This precise object recognition is essential for trait estimation at the organ level.

**Fig. 7:**
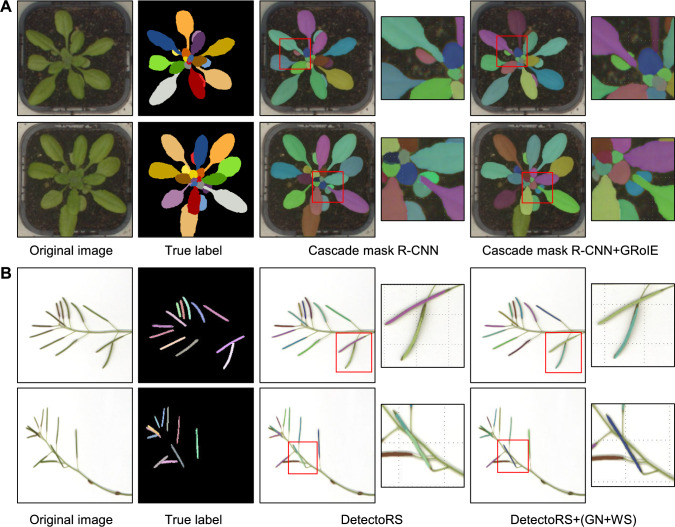
Instance segmentation of leaf and silique images using conventional computer vision models and the improved models. **A** Examples of leaf segmentation. From left to right: Two original images (typical RGB images); true labels (manually annotated labels for each individual leaf); output from Cascade Mask R-CNN (segmentation result using Cascade Mask R-CNN; the boxes zoom in on a difficult region); Cascade Mask R-CNN + GRoIE (segmentation results using the improved Cascade Mask R-CNN models, the boxes zoom in on the same difficult region). **B** Examples of silique segmentation results. From left to right: two original silique images, true labels for individual siliques, segmentation results from DetectoRS frame, with the boxes zooming in on overlapping siliques, segmentation results with our improved DetectoRS, with the boxes zooming in on overlapping siliques

To comprehensively demonstrate the algorithm's performance, we specifically selected two representative leaf images and two representative silique images as visualization cases (Fig. 7). A detailed examination of the processed images revealed that our method achieves superior segmentation results. Specifically, our improved model demonstrated enhanced discrimination capability, particularly with newly emerging leaves. For instance, in the second leaf image (Fig. 7, panel A), a leaf that was partly occluded by the petiole from an overlapping leaf was incorrectly segmented as two leaves by the original model, one on either side of the petiole of the overlapping leaf. The improved model incorporating GRoIE, however, produced accurate segmentation results, consistently outperforming the original model.

For silique images, the enhanced DetectoRS model provided more reliable segmentation results than the original model. A close-up view (Fig. 7B) illustrates the precise segmentation of overlapping siliques when the original model often resulted in only partial segmentation of many siliques, potentially compromising the accuracy of trait estimation. To quantify the performance of the segmentation models for leaves and siliques, we calculated the precision, recall, and F1 scores from all models (Table 2). The original Cascade Mask R-CNN model achieved precision, recall, and F1 score values of 0.956, 0.947, and 0.951, respectively, for leaf segmentation, whereas the improved model attained higher values for precision (0.965), recall (0.958), and F1 score (0.961). Similarly, for silique segmentation, the improved model incorporating GN and WS outperformed the original DetectoRS model, achieving higher precision and F1 score values of 0.954 (up from 0.944) and 0.942 (up from 0.921), respectively. These findings indicate that the improved models offer superior performance, leading to more accurate measurements of phenotypic traits.

### Generalizability of APTES to other datasets

To demonstrate its scalability and robustness, we applied APTES to three different Arabidopsis datasets: an Arabidopsis full-growth dataset, ARADEEPOPSIS, and the Plant Phenotyping Datasets (Fig. S1). APTES achieved excellent instance segmentation results across all tested datasets. Even in cases where the image resolution was low, as illustrated in Fig. S1C, all leaves were accurately segmented. These outcomes demonstrate that APTES possesses good generalizability and can be effectively applied to various Arabidopsis datasets collected under various environmental conditions.

### Generalizing to other plant species

**Fig. 8:**
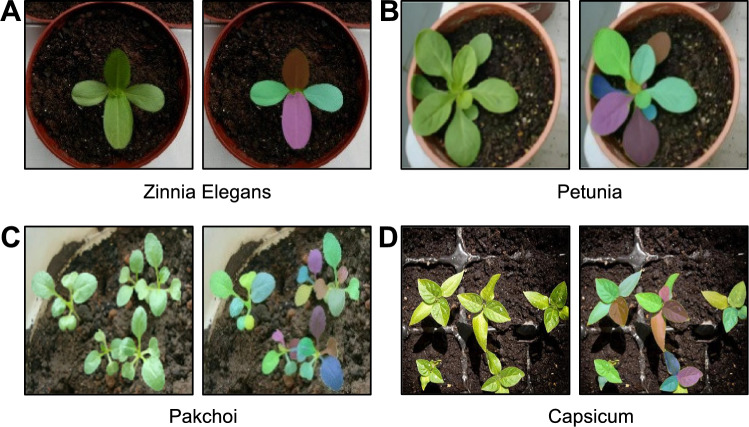
Segmentation results of individual leaves using the improved model for other plant species. **A** common zinnia (*Zinnia elegans*), **B** petunia (*Petunia*), **C** bok choy Chinese cabbage (*Brassica rapa* subsp. *chinensis*), and **D** pepper (*Capsicum* sp.)

Given the impressive independence of APTES from image resolution and growth conditions, we tested its generalization capacity for diverse plant species beyond Arabidopsis. To this end, we captured images of common zinnia (*Zinnia elegans*), petunia (*Petunia hybrida*), bok choy Chinese cabbage (*Brassica rapa* subsp. *chinensis*), and pepper (*Capsicum* sp.) with a smartphone camera and uploaded them to the APTES pipeline. The segmentation results for individual leaves are presented in Fig. 8. Our pipeline was successful in accurately distinguishing newly emerging leaves from adjacent ones in images with a single plant (Fig. 8A, B). APTES also correctly segmented all leaves in images containing multiple plants (Fig. 8C, D), demonstrating the ability of our pipeline to handle complex scenarios. Based on these segmentation results, APTES automatically computes traits for each leaf, underscoring its adaptability. The system measures morphometric traits in pixel units, which can be easily converted to metric measurements under controlled imaging conditions. For less controlled environments, such as those captured with smartphones, we advise including a scale object in the image to facilitate accurate metric conversions.

### Deep learning outperforms conventional methods in plant organ segmentation

Conventional segmentation methods encounter several limitations, such as the inability to precisely segment individual leaves or siliques from complex backgrounds when using thresholding techniques (as depicted in Fig. 4). These traditional approaches frequently lead to over-segmentation or under-segmentation, compromising the accuracy of trait measurements, which rely heavily on precise object segmentation. Additionally, their inefficiency poses a significant obstacle to their broad adoption.

However, with the widespread availability of GPUs, deep learning approaches utilizing CNNs have emerged as powerful tools for image segmentation. Advanced algorithms have been devised to harness the capabilities of CNNs to extract a wide array of local and global features from multiple layers, enabling a more comprehensive utilization of image data. As a result, CNNs have become the preferred option for image segmentation tasks. When sufficient labeled data are available, instance segmentation algorithms can effectively segment multiple objects within a single image, overcoming challenges associated with overlapping subjects and intricate backgrounds. We thus conducted an assessment of deep learning–based segmentation algorithms for the precise segmentation of individual plant organs within extensive datasets.

We developed APTES, an automated deep learning system that achieves high-precision segmentation and extracts 128 phenotypic traits from Arabidopsis with low measurement errors, demonstrating cross-species applicability. The newly developed computer vision APTES models have significantly improved the instance segmentation of Arabidopsis leaves and siliques, yielding a robust tool for the dissection of the genetic architecture underlying complex and dynamic morphometric traits. Nonetheless, certain constraints persist, such as the requirement to capture 2D images that reduce dimensional parameters (i.e., overhead image capture for leaf segmentation or post-harvest specimen preparation for silique segmentation). This limitation hinders the application of these models to data collected in less constrained or unconstrained environments, such as in the field. Fortunately, these models can be retrained (e.g., using active learning techniques) or potentially integrated with 3D modeling to address these issues.

The primary advantages of the APTES pipeline can be summarized as follows. (1) It represents the first instance segmentation method for individual leaves and siliques. Previous methods have primarily focused on segmenting entire Arabidopsis plants from their backgrounds, known as semantic segmentation. By contrast, the APTES approach enables the extraction of phenotypic parameters at the scale of single leaves or siliques, marking a significant advancement. (2) The system is highly practical and well-suited for Arabidopsis images obtained under various high-throughput phenotyping systems. We validated this capability using three publicly available datasets (Fig. 8). Furthermore, it supports leaf segmentation of single and multiple Arabidopsis plants, and the software can be directly applied to other plant species without the need for additional training. (3) The program and code are open-source, freely available for download and use by all researchers, with the aim of providing a practical and user-friendly tool for the Arabidopsis research community. (4) GWAS revealed 1,042 SNPs significantly associated with 18 leaf-related traits and a significant SNP on chromosome 3 associated with silique number, potentially involving the *OFT1* gene. In summary, we developed the APTES pipeline, which offers a valuable automated tool for leaf and silique segmentation and trait estimation that should benefit the broader plant science community.

### Limitations of APTES

APTES demonstrates robust performance for the segmentation of Arabidopsis leaves and siliques from plants grown under controlled laboratory conditions with uniform lighting and an absence of occlusions, but several critical limitations must be acknowledged regarding its practical application in agricultural settings. The model was trained exclusively on pot-grown Arabidopsis plants without environmental interference, which may lead to potentially compromised performance when using images collected under field conditions characterized by heterogeneous illumination, dynamic weather patterns, occluding vegetation, and complex backgrounds. Furthermore, the current pipeline exhibits limited generalizability to other plant species with divergent morphological characteristics, achieves lower segmentation accuracy under partial occlusion scenarios, and exhibits a substantial dependence on optimal image acquisition conditions. Additional constraints include unverified computational efficiency for large-scale deployment in real-time phenotyping systems and an absence of adaptive learning mechanisms to accommodate environmental variability. These limitations collectively highlight the need for expanded training datasets encompassing diverse field conditions, architecture enhancements for improved robustness against environmental noise, and integration of adaptive learning capabilities to facilitate broader agricultural applications. Future development should prioritize these aspects to enhance the practical utility of our model in real-world plant phenotyping scenarios.

Another limitation of the current research is that the APTES tool primarily provides phenotypic analysis at the morphological level. Although it can accurately quantify various geometric features of Arabidopsis plants, it does not establish a direct connection between these features and the underlying biochemical pathways that influence them, limiting an in-depth mechanistic explanation of phenotypic variation. To overcome this limitation, future research should explore multimodal correlations between morphological features and biochemical parameters (Zhao et al. 2022; Zhu et al. 2023). By integrating computational phenomics with metabolomics and other multidimensional data, a new, upgraded tool needs to be developed to connect macroscopic morphology to microscopic molecular mechanisms. This cross-scale research framework will help reveal phenotype–metabolite correlations that traditional single-dimensional analysis may overlook and provides new research ideas and technical tools for understanding the biochemical basis of plant morphology. We believe that through this methodological innovation, the APTES tool will evolve into an important bridge connecting plant phenomics and biochemical research.

## Materials and Methods

### A comprehensive software: APTES

The executable version of this software has been made available online for download to help meet the needs of Arabidopsis researchers. The software is accessible through a graphical user interface (GUI) that facilitates evaluation. Computers with GPU support can run the program directly. Detailed instructions for program execution can be found in the supplementary video. The initial GUI features three primary display windows: one for uploading the original image as input, another for downloading the individual segmented organs, and a third for downloading the measured phenotypic parameters. Segmented images can be exported to specific folders, and phenotypic parameters can be saved as Microsoft Excel spreadsheet files in default directories. Furthermore, the software supports processing in both single-image mode and batch mode for multiple images. All phenotypic parameters are reported as pixel units.

### An improved Cascade Mask R-CNN model for leaf segmentation: dataset, model, and processing flow

The Phenotyping Plant Datasets, provided by IPPN (Minervini et al. 2016), was initially utilized for our studies. This public image dataset contains only 285 images, which may potentially lead to overfitting when used for deep learning training. To mitigate this issue, data augmentation techniques such as Gaussian noise, color dithering, and rotation were applied to the original image dataset. Image stitching was employed as an additional data augmentation technique (Kuznichov et al. 2019). Following these data augmentation procedures, a total of 2000 images were generated. The final training dataset comprised three sets: (1) the original 250 leaf images, (2) 2000 augmented images generated from the original 250 images using conventional methods, and (3) 2000 stitched images created by the image stitching method. A total of 35 images were reserved for testing of the final model.

An improved Cascade Mask R-CNN was used for leaf segmentation, as illustrated in Fig. S2A. The performance of the model relies on the ability of the convolutional neural network (CNN) to extract features at different levels (Cai and Vasconcelos 2018). To enhance these capabilities, the backbone network was upgraded from a residual neural network (ResNet) to a residual split-attention network (ResNeSt), which is renowned for its improved feature representation. ResNeSt, owing to the adoption of group convolution and split-attention mechanisms, raises the complexity of the system model and strengthens the coupling between different dimensions of the parameter space, leading to better robustness and higher accuracy. Additionally, generic region of interest extraction (GRoIE) was integrated to refine the extraction of information from the region proposal network (RPN). Since each layer of the feature pyramid network (FPN) contains feature information, non-local construction modules and attention mechanisms were incorporated to extract more information. GRoIE incorporates multiscale features to minimize information loss and enhance the robustness and capability of feature acquisition (Fig. S2B) (Rossi et al. 2021). GRoIE can seamlessly integrate with the Cascade Mask R-CNN framework and exhibits superior performance compared to traditional region of interest (ROI) extractors. The framework for segmenting the leaf dataset is illustrated in Fig. S2.

### Improved DetectoRS model for silique segmentation: dataset, model, and processing flow

The seed pod images utilized in this study were obtained from the National Plant Phenomics Centre (NPPC) at Aberystwyth University, UK (Hamidinekoo et al. 2020). The plants were cultivated in pots of about 6 cm in diameter, using vermiculite to regulate air, moisture, and nutrient levels, along with a blend of sand, pea fertilizer, and stones to manage plant growth. Once they reached maturity, the inflorescences, stems, and siliques were harvested. The mature inflorescence or stem with attached fruits was harvested and scanned using a Plustek OpticPro A320 flatbed scanner (Shenzhen, China) and saved as *.png files with a resolution of 300 dots per inch (dpi). Each image had dimensions of 3600 × 5100 pixels, as exemplified in Fig. S3A. A dataset consisting of 160 images was compiled for silique segmentation and phenotypic trait analysis, with the number of siliques manually verified to assess accuracy.

Although the instance segmentation framework does not restrict the size of the input image, the dimensions of the image being processed significantly influence the power of the graphics card needed to support the analysis. In this scenario, using the original images directly for network training resulted in memory overflow on a 3080Ti graphics card. Therefore, the 160 original images were divided into 517 smaller images of varying sizes. Seed pod labeling was conducted using the LabelMe software (Russell et al. 2008), as depicted in Fig. S4B. To enhance the segmentation accuracy of the deep learning network, a total of 460 cropped images were augmented by applying horizontal flips, random scaling, and random clipping. In total, 1,840 augmented images were used for training, and 57 cropped images, without any augmentation, were used for testing.

To improve the accuracy and scalability of seed pod segmentation, this study refined an advanced deep learning model, DetectoRS, which is recognized for its competitive performance in instance segmentation tasks. DetectoRS incorporates a recursive feature pyramid (RFP) within the backbone network and includes additional feedback connections from FPN (Fig. S4B). In the RFP structure, the Atrous Spatial Pyramid Pooling (ASPP) module enriches feature representation, and the fusion module is responsible for integrating feature information from different stages. This design enhances the performance of the DetectoRS network in instance segmentation tasks, contributing to accurate estimation of seed pod parameters. DetectoRS also utilizes Switchable Atrous Convolution (SAC), a mechanism that has been demonstrated to enhance detector performance (Qiao et al. 2021). The architecture of the network is depicted in Fig. S4A.

Furthermore, whereas batch normalization (BN) is commonly used to optimize model training and mitigate the challenges associated with neural network training, larger batch sizes, which are typically advantageous for BN, were not feasible owing to hardware constraints, such as limited GPU memory capacity and computational resource limitations (Long et al. 2015). To address this issue, group normalization (GN) was adopted, which divides the features into multiple groups and normalizes them within each group. This approach ensures a more stable accuracy, even with fluctuating batch sizes. Additionally, weight standardization (WS) was implemented to refine weight optimization and accelerate neural network training (Qiao et al. 2019). Combining GN with WS was shown to significantly boost performance for tasks constrained by smaller batch sizes (Qiao et al. 2019). Consequently, our seed pod segmentation model integrated both GN and WS to overcome the limitations imposed by the small batch size.

### Performance evaluation of the improved models

In the classification task, four possible outcomes can describe the relationship between the true category and the category predicted by the classifier: True Positive (TP), False Positive (FP), False Negative (FN), and True Negative (TN). Specifically, a TP outcome arises when a true positive sample is correctly predicted as positive. An FP outcome occurs when a true negative sample is incorrectly predicted as positive. An FN outcome is the case where a true positive sample is incorrectly predicted as negative. Last, a TN outcome happens when a true negative sample is correctly predicted as negative.

To assess the performance of the instance segmentation model, metrics such as recall, precision, and the F1 score are commonly used. Each metric offers unique insights into model efficacy. Recall, also referred to as sensitivity or true positive rate, quantifies the proportion of true positive samples correctly identified by the model. Precision, also known as positive predictive value, measures the proportion of samples predicted as positive that are indeed positive. The F1 score, which combines both recall and precision into a single metric through their harmonic mean, provides a balanced evaluation that considers both precision and recall, particularly valuable in scenarios with uneven class distribution. The equations for these metrics are presented below.

1 $${\text{Precision}} = \frac{TP}{{TP + FP}}$$

2 $${\text{Recall}} = \frac{TP}{{TP + FN}}$$

3 $${\text{F}}1{\text{ score}} = \frac{2TP}{{2TP + FP + FN}}$$

In this study, the coefficient of determination (*R*^2^) and mean absolute percentage error (MAPE) were used to assess the performance of the model and the accuracy of phenotypic trait calculations. Specifically, MAPE, which is utilized to compare values obtained from the automated pipeline to those obtained from manual measurements, is defined in Eq. (4).

4 $${\text{MAPE}}\;{ = }\frac{1}{n}\sum\nolimits_{j = 1}^{n} {\frac{{\left| {x_{ai} - x_{mi} } \right|}}{{x_{mi} }}} \times 100\%$$

where ${x}_{mi}$ and ${x}_{ai}$ are manual measurement values and automated measurement values of the *i*th sample; *n* is the number of leaves or seed pods.

Furthermore, to evaluate the accuracy of the algorithm in determining the precise number of segmented organs, DiC was utilized to quantify the absolute discrepancy between the number of leaves or seed pods predicted by the algorithm (*#*L^p^*)* and the actual ground truth (#L^g^), as calculated with Eq. (5).

5 $${\text{DiC}} = \left| {\# {\text{L}}^{{\text{p}}} - \# {\text{L}}^{{\text{g}}} } \right|$$

### Trait calculations

For each segmented organ, APTES handles automated trait estimation before categorizing the phenotypic values into three distinct groups: morphometric, color, and texture traits. Instance segmentation is employed on images of Arabidopsis leaves and siliques, assigning each a unique ID for subsequent quantitative trait analysis. The estimation procedure for both leaf and seed pod traits adhered to a unified protocol. Morphological traits encompassed measures such as perimeter, area, circularity, and compactness, among others. Color traits offer insights into growth status by analyzing mean gray values and the distribution of various color components. Texture traits explore the surface patterns and variations of leaves and seed pods, including attributes such as roughness, contrast, directionality, line similarity, and regularity, thereby providing supplementary information on their visual structural characteristics. Notably, the system is equipped to compute a comprehensive set of 64 traits for leaves and an equivalent 64 traits for seed pods. The methodologies for calculating all phenotypic parameters were consistent across the leaf and seed pod analyses. The detailed description of all traits is given in Table S1.

### Genome-wide association study (GWAS)

A set of 166 Arabidopsis accessions from the RegMap panel (Horton et al. 2012) was grown and phenotyped covering the whole growth period using the APTES pipeline. Subsequently, the phenotypic data were used as input for GWAS on the GWAS-Portal platform (Seren 2018), relying on variant information for 250,000 single-nucleotide polymorphisms (SNPs) (Horton et al. 2012). The analysis was conducted based on a linear mixed model, and only SNPs with a minor allele frequency (MAF) exceeding 0.05 were considered. A Bonferroni correction was applied to account for multiple testing.

### Implementation details of the pipeline

Leaf and seed pod segmentation models were implemented within the PyTorch 1.9 framework, with the training step executed on an NVIDIA GeForce RTX 3080TI. For the leaf segmentation model, the network was trained for a total of 40 epochs using a stochastic gradient descent (SGD) optimizer, which featured a momentum of 0.9 and a weight decay of 0.00005. The initial learning rate was set to 0.001 and the batch size to 4. For the seed pod segmentation model, the network was trained for 24 epochs using the SGD optimizer, with a momentum of 0.9 and a weight decay of 0.0001. In this case, the initial learning rate was set to 0.0025 and the batch size to 2. Data augmentation was performed by random flipping, color dithering, and rotation. Detailed hyperparameters for both models are provided in Tables S6 and S7.

## Supplementary Information

Below is the link to the electronic supplementary material.

Supplementary file 1 (DOCX 1291 KB)

Supplementary file 2 (CSV 119 KB)

Supplementary file 3 (ZIP 44713 KB)

## Data Availability

The Plant Phenotyping Datasets were provided by IPPN and can be accessed at https://www.plant-phenotyping.org/datasets-home. The executable tool and software packages are available at https://drive.google.com/drive/folders/1i9IariiIrxuFtVIaRiaIzqvb8Gfg3xTc or http://plantphenomics.hzau.edu.cn/usercrop/Rice/download.
