# Supplementary material for: APTES: a high-throughput deep learning–based Arabidopsis phenotypic trait estimation system for individual leaves and siliques
Source: aBIOTECH. 2025 Oct 31;6(4):744–62. doi: 10.1007/s42994-025-00239-y (PMC12647460; doi:10.1007/s42994-025-00239-y)
Supplement: Supplementary file 1 — (DOCX 1291 KB) [file 42994_2025_239_MOESM1_ESM.docx]

## Supplementary Data files

The supplementary data files can be accessed at https://drive.google.com/drive/folders/1i9IariiIrxuFtVIaRiaIzqvb8Gfg3xTc or <http://plantphenomics.hzau.edu.cn/usercrop/Rice/download.>

1. Fig. S1: Testing APTES on three independent datasets
2. Fig. S2: Generic region of interest extraction (GRoIE) structure and network architecture of Cascade Mask R-CNN
3. Fig. S3: Silique images and labeling of individual seed pods
4. Fig. S4: Network architecture and recursive feature pyramid (RFP) structure of DetectoRS
5. Table S1: Trait calculation methods
6. Table S2: List of SNPs used for the GWAS (in Excel)
7. Table S3: Hardware environment
8. Table S4: Software environment
9. Table S5: Evaluation results of image segmentation efficiency for different sizes of leaves and seed pods
10. Table S6: Overview of hyperparameter configurations for leaf segmentation
11. Table S7: Overview of hyperparameter configurations for seed pod segmentation
12. Supplementary Video: How to use the ATPES software


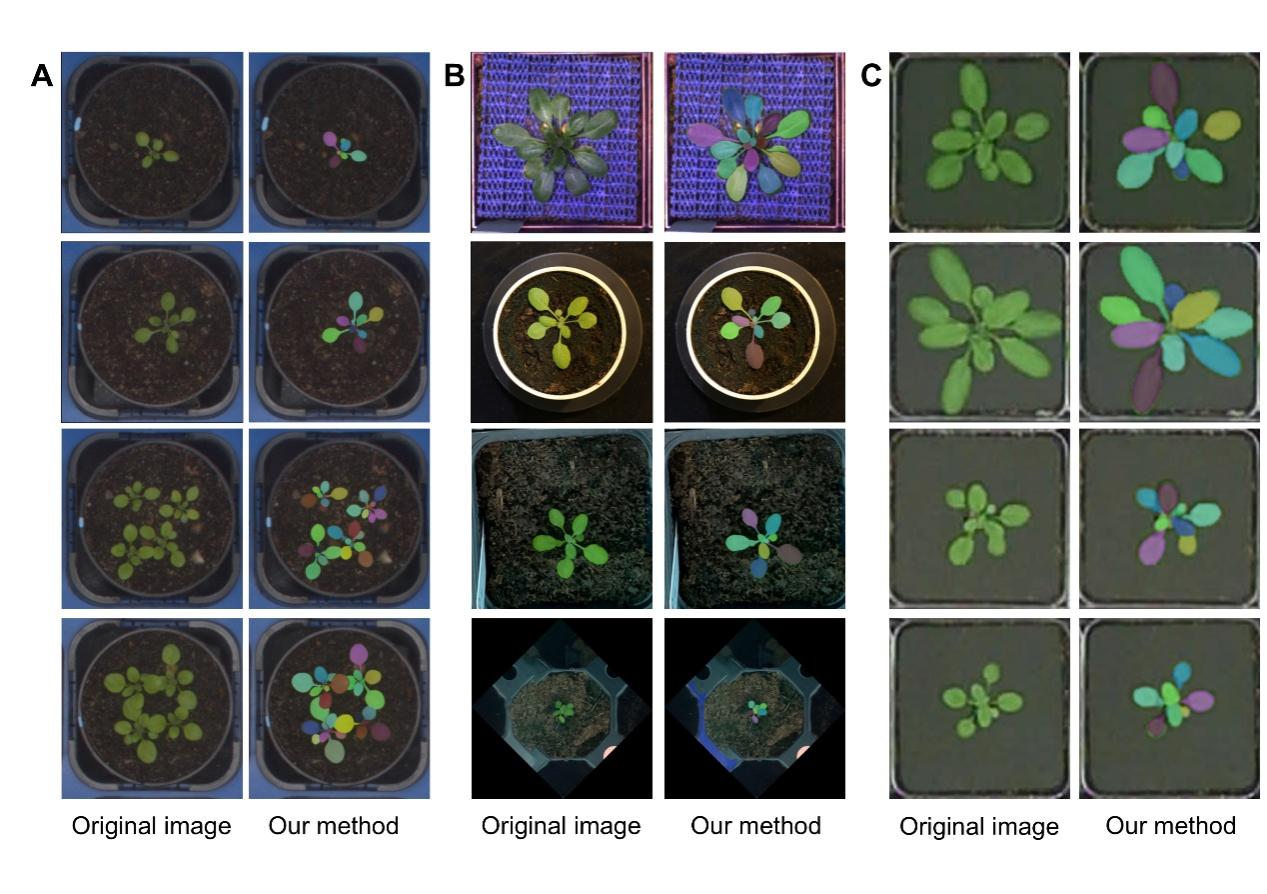


**FigS1.** Testing APTES on three independent datasets. **A** Arabidopsis full-growth dataset; **B** Arabidopsis dataset; and **C** Plant Phenotyping Datasets. Left, original images; right, segmented results.


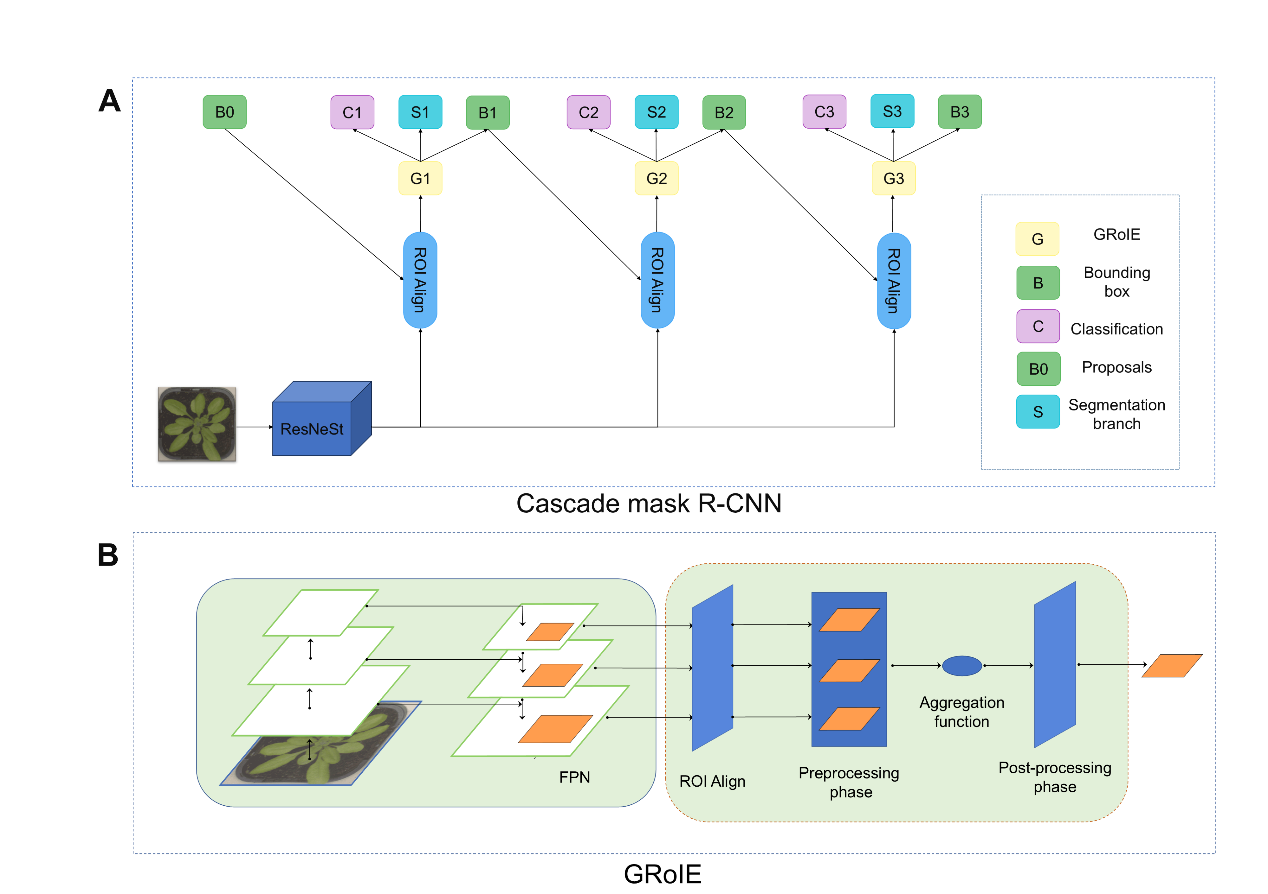


**Fig. S2** Generic region of interest extraction (GRoIE) structure and network architecture of Cascade Mask R-CNN. A Diagram illustrating the four modules of the GRoIE structure. The FPN module is a feature pyramid network architecture used for object detection and semantic segmentation. The ROI Align module performs maximum pooling on non-uniform regions of interest (ROIs) to produce a fixed-size representation. The Pre-processing module applies preliminary processing to the pooled regions. The Aggregation module determines how to combine the individual ROIs from each branch. The Post-processing module applies additional processing to the merged features before they are returned. B Diagram of the Cascade Mask R-CNN network architecture. Here, GRoIE represents the entire diagram shown in (**A**). Proposals are generated by the region proposal network (RPN) structure. ResNeSt101 is employed as the backbone network.


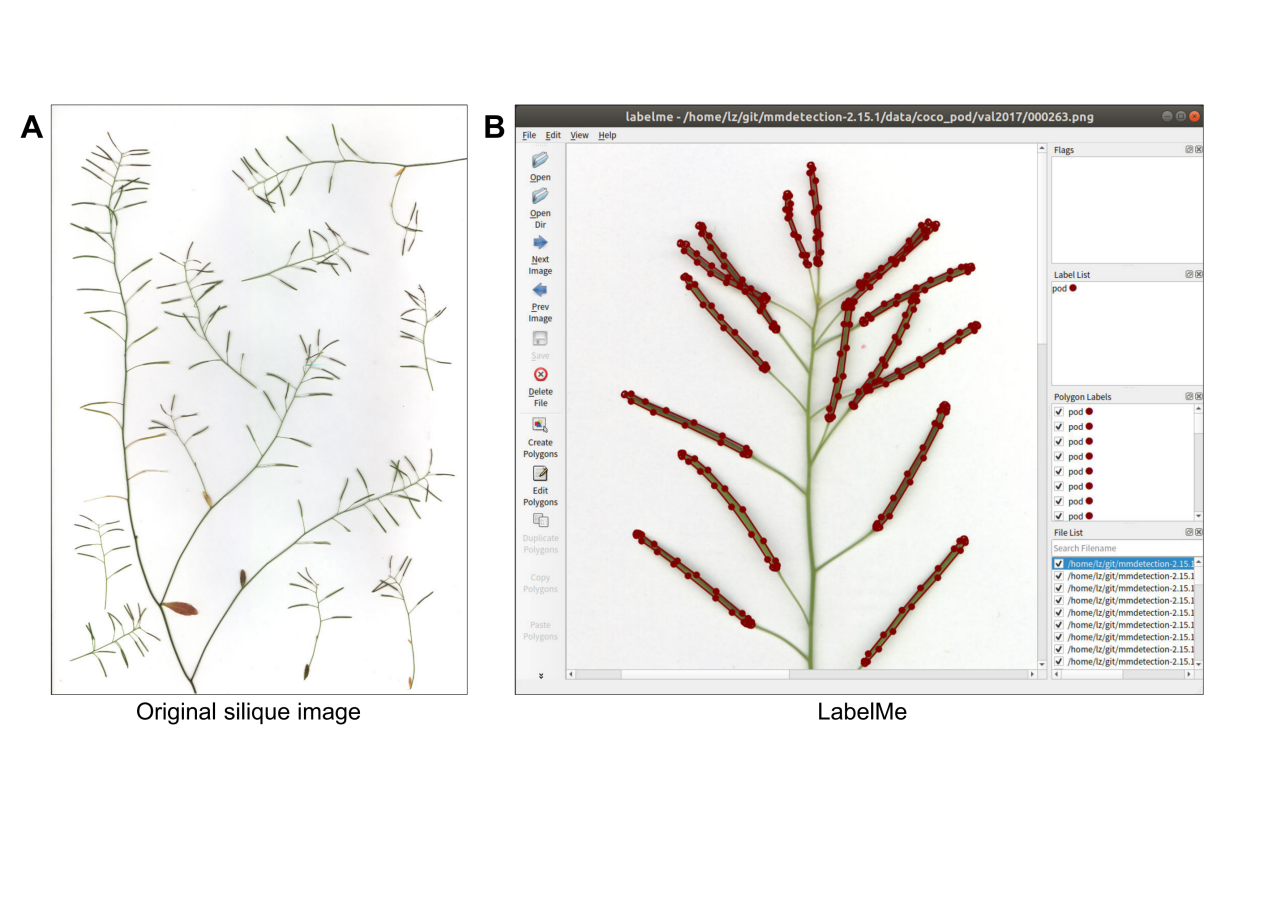


**Fig. S3** Generic region of interest extraction (GRoIE) structure and network architecture of Cascade Mask R-CNN. **A** Diagram illustrating the four modules of the GRoIE structure. The FPN module is a feature pyramid network architecture used for object detection and semantic segmentation. The ROI Align module performs maximum pooling on non-uniform regions of interest (ROIs) to produce a fixed-size representation. The Pre-processing module applies preliminary processing to the pooled regions. The Aggregation module determines how to combine the individual ROIs from each branch. The Post-processing module applies additional processing to the merged features before they are returned. **B** Diagram of the Cascade Mask R-CNN network architecture. Here, GRoIE represents the entire diagram shown in (A). Proposals are generated by the region proposal network (RPN) structure. ResNeSt101 is employed as the backbone network. B, classification task branch; C, regression task branch; and S, semantic segmentation task branch.


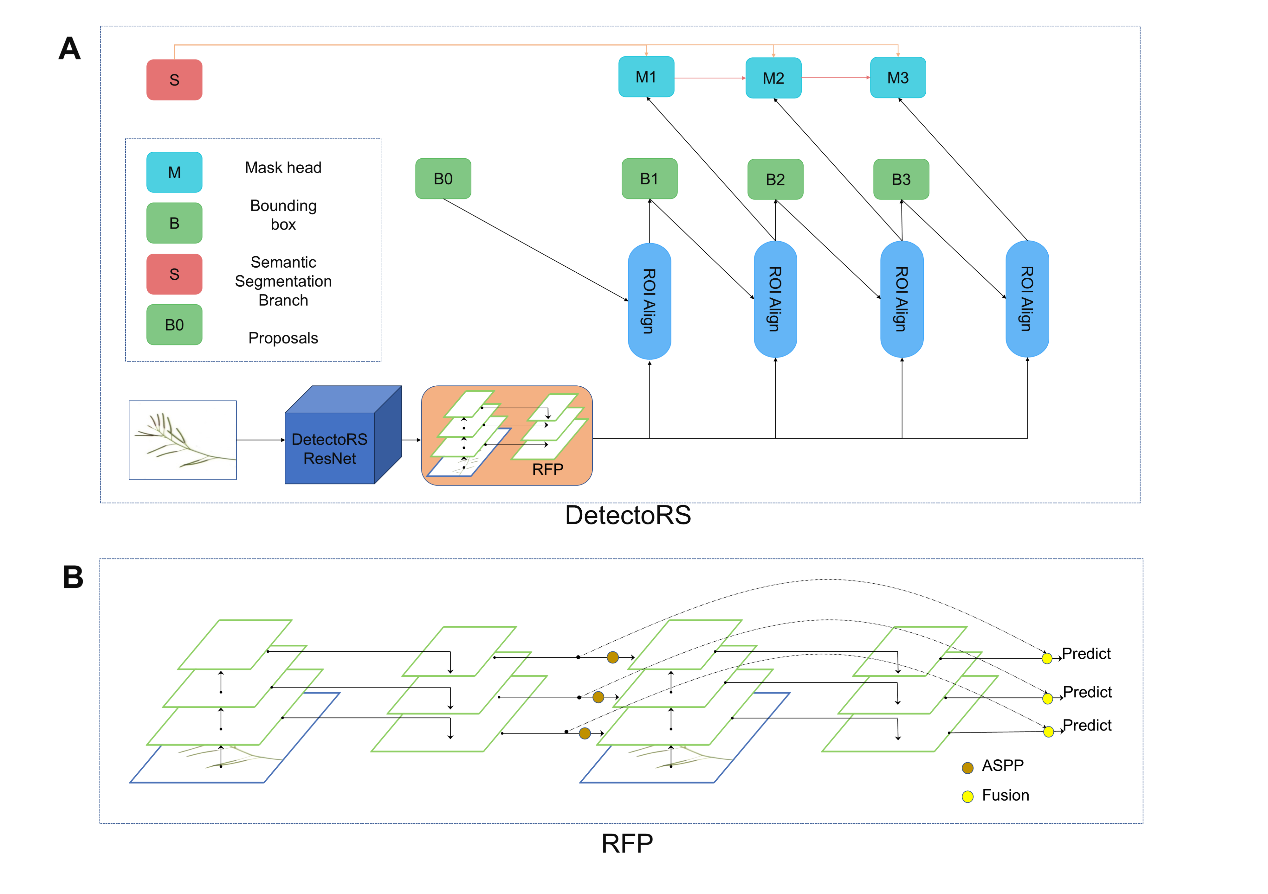


**Fig. S4** Network architecture and recursive feature pyramid (RFP) structure of DetectoRS. **A** Diagram of the DetectoRS network architecture. The RFP structure is indicated in (**B**). Proposals are generated by the region proposal network (RPN) structure. ResNeSt101 is utilized as the backbone network. **B**, task branch for the generation of bounding boxes; S, task branch for the generation of semantic segmentation. B Diagram of the RFP structure, which employs the Atrous Spatial Pyramid Pooling (ASPP) module to enrich feature representations and a fusion module to combine features from different processing stages.

Supplementary Table S1. Trait calculation methods

| **Group** | **Description** | **Acronym** | **Equation** |  |
| --- | --- | --- | --- | --- |
| Morphological  Traits | It represents the length of the outline of a single leaf. Perimeter is quantified by counting the number of pixels that form the boundary of the leaf or pod, denoted as P. | P | / | 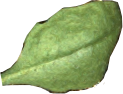  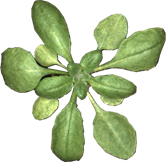 |
|  | It represents the average perimeter of all the leaves of a single Arabidopsis plant. | Average Perimeter | $Average\_Perimeter=\frac{\sum_{i=1}^{n} P_{i}}{n}$ |  |
|  | It represents the maximum perimeter of the leaves of a single Arabidopsis plant. | Maximum Perimeter | $Maximum Perimeter=max(P_{i})$ |  |
|  | It represents the standard deviation of the perimeters of all the leaves of a single Arabidopsis plant. | STD_Perimeter | $STD\_Perimeter=\sqrt{\frac{\sum_{i=1}^{n} {(P_{i}-Mean\_Perimeter)}^{2}}{n}}$ |  |
|  | It represents the coefficient of variation (CV) of the perimeters of all the leaves of a single Arabidopsis plant. | CV_Perimeter | $CV\_Perimeter=\frac{STD\_Perimeter}{Average Perimeter}$ |  |
|  | Roundness is a geometric parameter that describes how close a shape is to a perfect circle. This ratio reflects the smoothness and regularity of a shape. The value of roundness ranges from 0 to 1, where 1 represents a perfect circle. | Roundness | $Roundness=\frac{4\pi S}{P^{2}}$ |  |
|  | It represents the average roundness of all the leaves of a single Arabidopsis plant. | Average Roundness | *Average Roundness*$=\frac{\sum_{i=1}^{n} C_{i}}{n}$ |  |
|  | It represents the maximum roundness of all leaves | Maximum Roundness | $Maximum Roundness=max(C_{i})$ |  |
|  | It represents the standard deviation of the roundness of all leaves. | STD_Roundness | $STD\_Roundness=\sqrt{\frac{\sum_{i=1}^{n} {(C_{i}-Average Roundness)}^{2}}{n}}$ |  |
|  | It represents the coefficient of variation (CV) the roundness of all leaves. | CV_Roundness | $CV\_Roundness=\frac{STD\_Roundness}{Average Roundness}$ |  |
|  | The number of leaves on a single Arabidopsis plant. | Total Leaf Number | / |  |
|  | Area represents the overall size of a single leaf or pod organ. It is calculated by counting the number of pixels that are contained within the boundary of the leaf or pod, and is denoted as S. | S | / |  |
|  | It represents the total area of all the leaves of each individual Arabidopsis plant. | Total Leaf Area | $Total Leaf Area=\sum_{i=1}^{n} S_{i}$ |  |
|  | It represents the average area of all the leaves of a single Arabidopsis plant. | Average Leaf Area | $Average Leaf Area=\frac{\sum_{i=1}^{n} S_{i}}{n}$ |  |
|  | It represents the maximum area of all the leaves of a single Arabidopsis plant. | Maximum Leaf Area | $Maximum Leaf Area=max(S_{i})$ |  |
|  | It represents the standard deviation of the leaf area distribution of a single Arabidopsis plant. | STD_Area | $STD\_Area=\sqrt{\frac{\sum_{i=1}^{n} {(S_{i}-Average Leaf Area)}^{2}}{n}}$ |  |
|  | It represents the coefficient of variation (CV) for the area of leaves. | CV_Area | $CV\_Area=\frac{STD\_Area}{Average Leaf Area}$ |  |
|  |  |  |  |  |
|  | The orthogonal bounding box is used to describe the rectangular box that can completely contain a leaf. This box is aligned with the coordinate axes, meaning its boundaries are parallel to the X-axis and Y-axis. | OBB | / | 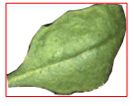 |
|  | Leaf length ori is defined as the length of the orthogonal bounding rectangle. | Leaf length ori | / |  |
|  | Leaf width ori is defined as the width of the orthogonal bounding rectangle. | Leaf width ori | / |  |
|  | Average length_ori is defined as the average values of all the leaf lengths (Leaf length ori) of a single Arabidopsis plant. | Average length_ori | $Average length\_ori=\frac{\sum_{i=1}^{n} {Leaf length ori}_{i}}{n}$ |  |
|  | Average width_ori is defined as the average values of all the leaf widths (Leaf width ori) of a single Arabidopsis plant. | Average width_ori | $Average width\_ori=\frac{\sum_{i=1}^{n} {Leaf width ori}_{i}}{n}$ |  |
|  | C_max_ori is defined as the maximum length of the orthogonal bounding rectangle. | c_max_ori | *c_max_ori=*$max({Leaf length ori}_{i})$ |  |
|  | H_max_ori is defined as the maximum width of the orthogonal bounding rectangle. | h_max_ori | *h_max_ori=*$max({Leaf width ori}_{i})$ |  |
|  |  |  |  |  |
|  | The minimum bounding box is the smallest rectangle that can encompass a set of points, often used to describe the position, size, and orientation. | MBB | / | 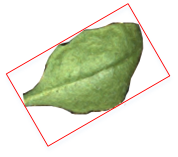 |
|  | The area of the minimum bounding box refers to the area value of this minimum bounding box. | Area_MBB | / |  |
|  | Leaf length is defined as the length of the minimum bounding rectangle. | d | / |  |
|  | Leaf width is defined as the width of the minimum bounding rectangle. | e | / |  |
|  | Average Length is defined as the average values of all leaf lengths of a single Arabidopsis plant. | Average Length | $Average Length=\frac{\sum_{i=1}^{n} d_{i}}{n}$ |  |
|  | It represents the maximum length of the minimum bounding rectangles of all the leaves of a single Arabidopsis plant. | Maximum Length | $Maximum Length=max(d_{i})$ |  |
|  | It represents the standard deviation of the lengths of the minimum bounding rectangles of all the leaves of a single Arabidopsis plant. | STD_Length | *STD_Length*  $=\sqrt{\frac{\sum_{i=1}^{n} {(d_{i}-Average Length)}^{2}}{n}}$ |  |
|  | It represents the coefficient of variation (CV) of the lengths of the minimum bounding rectangles of all the leaves of a single Arabidopsis plant. | CV_Length | $CV\_Length=\frac{STD\_Length}{Average Length}$ |  |
|  | Average Width is defined as the average values of all leaf widths of a single Arabidopsis. | Average Width | $Average Width=\frac{\sum_{i=1}^{n} e_{i}}{n}$ |  |
|  | It represents the maximum width of the minimum bounding rectangles of all the leaves of a single Arabidopsis plant. | Maximum Width | $Maximum Width=max(e_{i})$ |  |
|  | It represents the standard deviation of the widths of the minimum bounding rectangles of all the leaves of a single Arabidopsis plant. | STD_Width | $STD\_Width=\sqrt{\frac{\sum_{i=1}^{n} {(e_{i}-Average Width)}^{2}}{n}}$ |  |
|  | It represents the coefficient of variation (CV) of the widths of the minimum bounding rectangles of all the leaves of a single Arabidopsis plant. | CV_Width | $CV\_Width=\frac{STD\_Width}{Average Width}$ |  |
|  | It represents the average area of the minimum bounding box of all leaves for a single Arabidopsis plant. | Average MBB_Area | $Average MBB\_Area=\frac{\sum_{i=1}^{n} d_{i}*e_{i}}{n}$ |  |
|  | It represents the maximum area of the minimum bounding rectangles of all the leaves of a single Arabidopsis plant. | MBB_Area | $MBB\_Area=\max\left( d_{i}*e_{i} \right), (i=1,2,\ldots,n)$ |  |
|  | It represents the standard deviation of the areas of the minimum bounding rectangles of all the leaves of a single Arabidopsis plant. | STD_MBB_Area | $STD\_MBB\_Area=\sqrt{\frac{\sum_{i=1}^{n} {(d_{i}*e_{i}-Average MBB\_Area)}^{2}}{n}}$ |  |
|  | It represents the coefficient of variation (CV) of the areas of the minimum bounding rectangles of all the leaves of a single Arabidopsis plant. | CV_MBB_Area | $CV\_MBB\_Area=\frac{STD\_MBB\_Area}{Average MBB\_Area}$ |  |
|  | Average Area/MBB_Area represents the ratio of the average area of all leaves to the area of the minimum bounding rectangles of the leaves for one single Arabidopsis plant. It refers to the degree to which a leaf resembles a rectangle. | Average Area/MBB_Area | $Average Area/MBB\_Area=\frac{Average Area}{Average MBB\_Area}$ |  |
|  | It represents the standard deviation of the average area of all leaves to the area of the minimum bounding rectangles of the leaves for one single Arabidopsis plant. | STD_Area/MBB_Area | $STD\_Area/MBB\_Area=\sqrt{\frac{\sum_{i=1}^{n} {({Area/MBB\_Area}_{i}-Average Area/MBB\_Area)}^{2}}{n}}$ |  |
|  | It represents the ratio of the average perimeter to the average area of the leaves of a single Arabidopsis plant. This ratio helps in understanding the compactness of a shape, with higher values indicating less compact shapes. | Average Perimeter/Area | $Average Perimeter/Area=\frac{Average Perimeter}{Average Leaf Area}$ |  |
|  | It represents the standard deviation of the average perimeter to the mean area of the leaves of a single Arabidopsis plant. | STD_Perimeter/Area | $STD\_Perimeter/Area=\sqrt{\frac{\sum_{i=1}^{n} {({P/S}_{i}-Average Area/MBB\_Area)}^{2}}{n}}$ |  |
|  | The convex hull of a leaf is the smallest convex polygon that can contain the geometric shape. The area of the convex hull refers to the area of this smallest convex polygon. | Convex Hull Leaf Area | / |  |
|  |  |  |  |  |
| Color traits | It represents the mean value of the red channel for all the leaves of a single Arabidopsis plant. | R_Ratio_mean | $R\_Ratio\_mean=\frac{\sum_{i=1}^{n} R_{i}}{n}$ |  |
|  | It represents the standard deviation of the red channel for all the leaves of a single Arabidopsis plant. | STD_R_mean | $STD\_R\_mean=\sqrt{\frac{\sum_{i=1}^{n} {(R_{i}-R\_Ratio\_mean)}^{2}}{n}}$ |  |
|  | It represents the mean value of the green channel for all the leaves of a single Arabidopsis plant. | G_Ratio_mean | $G\_Ratio\_mean=\frac{\sum_{i=1}^{n} G_{i}}{n}$ |  |
|  | It represents the standard deviation of the green channel for all the leaves of a single Arabidopsis plant. | STD_G_mean | $STD\_G\_mean=\sqrt{\frac{\sum_{i=1}^{n} {(G_{i}-G\_Ratio\_mean)}^{2}}{n}}$ |  |
|  | It represents the mean value of the blue channel for all the leaves of a single Arabidopsis plant. | B_Ratio_mean | $B\_Ratio\_mean=\frac{\sum_{i=1}^{n} B_{i}}{n}$ |  |
|  | It represents the standard deviation of the blue channel for all the leaves of a single Arabidopsis plant. | STD_B_mean | $STD\_B\_mean=\sqrt{\frac{\sum_{i=1}^{n} {(B_{i}-B\_Ratio\_mean)}^{2}}{n}}$ |  |
|  | It represents the proportion of the green component for a single Arabidopsis plant. | G_All_mean | $G\_All\_mean=\frac{G}{R+G+B}$ |  |
| Texture Traits | Gray gradient co-occurrence matrix is a method of extracting image texture features by statistically analyzing the co-occurrence relationship between the gray values and gradient values of pixels in an image.  The 15 gray level co-occurrence matrix texture traits, including the correlation (T1), the advantages of the small gradient (T2), the advantages of the large gradient (T3), the energy (T4), the intensity inhomogeneity (T5), the gradient inhomogeneity (T6), the mean gray (T7), the mean gradient (T8), the gray entropy (T9), the gradient entropy (T10), the entropy of mixing (T11), the differential moment (T12), the deficit score (T13), the gray variance (T14), and the gradient variance (T15). | Gray-Gradient  Co-occurrence Matrix | $T1=\frac{1}{\sigma_{1}\sigma_{2}}\sum_{i=1}^{32} \sum_{j=1}^{32} (i-\mu_{1})(j-\mu_{2})P(i,j)$ $T2=\sum_{i=1}^{32} \sum_{j=1}^{32} \frac{P(i,j)}{j^{2}}/\sum_{i=1}^{32} \sum_{j=1}^{32} P(i,j)$ $T3=\sum_{i=1}^{32} \sum_{j=1}^{32} j^{2}P(i,j)/\sum_{i=1}^{32} \sum_{j=1}^{32} P(i,j)$ $T4=\sum_{i=1}^{32} \sum_{j=1}^{32} P(i,j)^{2}$ $T5=\sum_{i=1}^{32} [\sum_{j=1}^{32} P(i,j)]^{2}/\sum_{i=1}^{32} \sum_{j=1}^{32} P(i,j)$ $T6=\sum_{j=1}^{32} [\sum_{i=1}^{32} P(i,j)]^{2}/\sum_{i=1}^{32} \sum_{j=1}^{32} P(i,j)$ $T7=\mu_{1}$ $T8=\mu_{2}$ $T9=-\sum_{i=1}^{32} \sum_{j=1}^{32} P(i,j)\cdot\log\sum_{j=1}^{32} P(i,j)$ $T10=-\sum_{j=1}^{32} \sum_{i=1}^{32} P(i,j)\cdot\log\sum_{i=1}^{32} P(i,j)$ $T11=-\sum_{i=1}^{32} \sum_{j=1}^{32} P(i,j)\cdot\log P(i,j)$ $T12=\sum_{i=1}^{32} \sum_{j=1}^{32} (i-j)^{2}P(i,j)$ $T13=\sum_{i=1}^{32} \sum_{j=1}^{32} \frac{1}{1+(i-j)^{2}}P(i,j)$ $T14=\sigma_{1}$ $T15=\sigma_{2}$ $\mu_{1}=\sum_{i=1}^{32} i\cdot\sum_{j=1}^{32} P(i,j)$ $\mu_{2}=\sum_{j=1}^{32} j\cdot\sum_{i=1}^{32} P(i,j)$ $\sigma_{1}=\sqrt{\sum_{i=1}^{32} (i-\mu_{1})^{2}\sum_{j=1}^{32} P(i,j)}$ $\sigma_{2}=\sqrt{\sum_{j=1}^{32} (j-\mu_{2})^{2}\sum_{i=1}^{32} P(i,j)}$                                      |  |
| Other  Traits  (Geometric Traits) | It represents a set of seven numerical values derived from the geometric moments of an image. | Hu moments1-7 | Hu's invariant moments are based on the normalized central moments of the image, where $\mu_{pq}$ is defined as:  $\mu_{pq}=\sum_{x,y} (x-x)^{p}(y-y)^{q}f(x,y)$  Here, $(x,y)$ are the coordinates of the image centroid, and $f(x,y)$ is the intensity of the image at the coordinates $(x,y)$.  Then, from these central moments, the normalized central moments $\eta_{ij}$ can be computed as:$\eta_{ij}=\frac{\mu_{ij}}{\mu_{00}^{(1+(i+j)/2)}}$  where $\mu_{00}$ is the zeroth order central moment of the image.  $I1=\eta_{20}+\eta_{20}$  $I2={(\eta_{20}-\eta_{02})}^{2}+4\eta_{11}^{2}$  $I3={(\eta_{30}-3\eta_{12})}^{2}+{({3\eta}_{21}-\eta_{03})}^{2}$  $I4={(\eta_{30}+\eta_{12})}^{2}+{(\eta_{21}+\eta_{03})}^{2}$  $I5=(\eta_{30}-3\eta_{12})(\eta_{30}+\eta_{12})[{(\eta_{30}+\eta_{12})}^{2}-3{(\eta_{21}+\eta_{03})}^{2}]+({3\eta}_{21}-\eta_{03})(\eta_{21}+\eta_{03})[3{(\eta_{30}+\eta_{12})}^{2}-{(\eta_{21}+\eta_{03})}^{2}]$  $I6=(\eta_{20}-\eta_{02})[{(\eta_{30}+\eta_{12})}^{2}-{(\eta_{21}+\eta_{03})}^{2}]+4\eta_{11}(\eta_{30}+\eta_{12})(\eta_{03}+\eta_{21})$  $I7=(3\eta_{21}-\eta_{03})(\eta_{30}+\eta_{12})[{(\eta_{30}+\eta_{12})}^{2}-3{(\eta_{21}+\eta_{03})}^{2}]-(\eta_{30}-3\eta_{12})(\eta_{30}+\eta_{12})[{3(\eta_{30}+\eta_{12})}^{2}-{(\eta_{21}+\eta_{03})}^{2}]$ |  |

Supplementary Table S**3. Hardware environment**

| Hardware Component | Model | Quantity |
| --- | --- | --- |
| Processor | AMD 5800X | 1 |
| Memory | 32GB | 2 |
| GPU | NVIDIA GeForce RTX 3090 | 1 |

Supplementary Table S4. Software environment

| Software Component | Version/Details |
| --- | --- |
| Operating System | Ubuntu 20.04 |
| MMDetection | 2.16.0 |
| Pytorch | 1.8.0 |
| Python | 3.7 |
| Cuda | 11.1 |
| Cudnn | 8.5.0 |
| Opencv | 4.5.4 |
| Mmcv-full | 1.4.6 |

Supplementary Table S5. Evaluation Results of Image Segmentation Efficiency for Different Sizes of Leaves and Pod

| Image Size (Leaf) | Time/s | Image Size (Pod) | Time/s |
| --- | --- | --- | --- |
| 92.94KB, 218x226 pixels | 0.614 | 28.2KB, 817x793 pixels | 1.189 |
| 274.34KB, 441x441 pixels | 1.476 | 70.0KB, 1525x1113 pixels | 3.256 |

Supplementary Table S6. **Overview of hyperparameter configurations for leaf segmentation**

| **Parameter Name** | **Value** |
| --- | --- |
| IMAGE_SIZE | 1333×640, 1333×800 |
| BASE_LR | 1e-2 |
| WEIGHT_DECAY | 0.0001 |
| MOMENTUM | 0.9 |
| WARMUP_ITERS | 500 |
| WARMUP_RATIO | 0.001 |
| EPOCHS | 20 |
| POST_IOU_THR | 0.5, 0.6, 0.7 |
| NEGATIVE_IOU_THR | 0.5, 0.6, 0.7 |
| SOFT_IOU_THRESHOLD | 0.46 |
| BATCH SIZE | 2 |

Supplementary Table S7. Overview of hyperparameter configurations for seed pod segmentation

| **Parameter Name** | **Value** |
| --- | --- |
| IMAGE_SIZE | 1333×800 |
| BATCH SIZE | 2 |
| EPOCHS | 72 |
| MOMENTUM | 0.9 |
| WARMUP_ITERS | 500 |
| WARMUP_RATIO | 0.001 |
| BASE_LR | 1e-2 |
| WEIRHT_DECAY | 0.0001 |
| SOFT_IOU_THRESHOLD | 0.48 |
| POST_IOU_THR | 0.5，0.6，0.7 |
| NSETIVE_IOU_THR | 0.5，0.6，0.7 |
